# Brazilian Dialysis Census: analysis of data from the 2009-2018 decade

**DOI:** 10.1590/2175-8239-JBN-2019-0234

**Published:** 2020-05-20

**Authors:** Precil Diego Miranda de Menezes Neves, Ricardo de Castro Cintra Sesso, Fernando Saldanha Thomé, Jocemir Ronaldo Lugon, Marcelo Mazza Nasicmento

**Affiliations:** (1) Universidade de São Paulo, Faculdade de Medicina, Hospital das Clínicas, São Paulo, SP, Brasil.; 2 Hospital Alemão Oswaldo Cruz, São Paulo, SP, Brasil.; 3 Universidade Federal de São Paulo, São Paulo, SP, Brasil.; 4 Universidade Federal do Rio Grande do Sul, Porto Alegre, RS, Brasil.; 5 Universidade Federal Fluminense, Niterói, RJ, Brasil.; 6 Universidade Federal do Paraná, Curitiba, PR, Brasil.

**Keywords:** Censuses, Kidney Failure, Chronic, Epidemiology, Renal Dialysis, Peritoneal Dialysis, Brazil, Censos, Falência Renal Crônica, Epidemiologia, Diálise Renal, Diálise Peritoneal, Brasil

## Abstract

**Introduction:**

National data on chronic dialysis treatment are essential for the development of health policies that aim to improve patient treatment.

**Objective:**

To present data from the Brazilian Society of Nephrology on patients with chronic dialysis for kidney disease in July 2018, making a comparative analysis of the past 10 years.

**Methods:**

Data collection from dialysis units, with filling in an online questionnaire for 2018. Data from 2009, 2013 and 2018 were compared.

**Results:**

288 (36.6%) centers answered the questionnaire. In July 2018, the estimated total number of patients on dialysis was 133,464. Estimates of the prevalence and incidence rates of patients undergoing dialysis treatment per million of the population (pmp) were 640 and 204, respectively, with average annual increases of 23.5 pmp and 6 pmp for prevalence and incidence, respectively. The annual gross mortality rate was 19.5%. Of the prevalent patients, 92.3% were on hemodialysis and 7.7% on peritoneal dialysis, with 29,545 (22.1%) on the waiting list for transplantation. Median bicarbonate concentration in the hemodialysis bath was 32 mEq/L. Venous catheters were used as access in 23.6% of the hemodialysis patients. The prevalence rate of positive serology for hepatitis C showed a progressive reduction (3.2%).

**Conclusion:**

The absolute number of patients and rates of incidence and prevalence in dialysis in the country increased substantially in the period, although there are considerable differences in rates by state. There has been a persistent increase in the use of venous catheters as an access for dialysis; and reduction in the number of patients with positive serology for hepatitis C.

## RESUMO

**Introdução:**

Dados nacionais sobre o tratamento dialítico crônico são essenciais para a elaboração de políticas de saúde que almejem melhora no tratamento dos pacientes.

**Objetivo:**

Apresentar dados do inquérito da Sociedade Brasileira de Nefrologia sobre pacientes com doença renal crônica dialítica em julho de 2018, fazendo análise comparativa dos últimos 10 anos.

**Métodos:**

Coleta de dados a partir das unidades de diálise, com preenchimento de questionário on-line referentes a 2018. Foram comparados dados de 2009, 2013 e 2018.

**Resultados:**

288 (36,6%) centros responderam ao questionário. Em julho de 2018, o número total estimado de pacientes em diálise foi de 133.464. Estimativas das taxas de prevalência e incidência de pacientes em tratamento dialítico por milhão da população (pmp) foram de 640 e 204, respectivamente, com médias de aumento anuais de 23,5 pmp e 6 pmp para prevalência e incidência, respectivamente. Taxa anual de mortalidade bruta foi de 19,5%. Dos pacientes prevalentes, 92,3% estavam em hemodiálise e 7,7%, em diálise peritoneal, com 29.545 (22,1%) em fila de espera para transplante. Concentração mediana de bicarbonato no banho de hemodiálise foi de 32 mEq/L. Cateter venoso era usado como acesso em 23,6% dos pacientes em hemodiálise. Taxa de prevalência de sorologia positiva para hepatite C apresentou redução progressiva (3,2%).

**Conclusão:**

Número absoluto de pacientes e taxas de incidência e prevalência em diálise no país aumentaram substancialmente no período, embora haja diferenças consideráveis nas taxas por estado. Tem havido persistente aumento no uso de cateter venoso como acesso para diálise; e redução de pacientes com sorologia positiva para hepatite C.

## INTRODUCTION

For the tenth consecutive year, the Brazilian Society of Nephrology (SBN) conducts the Brazilian Census on Dialysis.^1^^-^ 8 This is a nationwide online survey, aimed at gathering information on patients undergoing chronic dialysis in the centers of registered active dialysis. The epidemiological and technical data gathered through this census are important health policy tools, enabling, in addition to knowing the profile of patients, the formulation of projects and strategies that improve their care. Despite the problems inherent to research based on voluntary data provision, a significant portion of kidney care centers in Brazil has contributed to this initiative.

This paper compares the clinical and laboratory profile of patients on chronic dialysis in Brazil in the past 10 years (2009-2018), seeking to show trends in the variation of characteristics evaluated in this long period of chronic dialysis treatment in the country.

## METHODS

### DATA COLLECTION

During the second semester of 2018, we carried out a survey in the dialysis centers registered with the Brazilian Society of Nephrology in order to collect and analyze the data of patients undergoing outpatient chronic renal replacement therapy. To this end, a questionnaire with questions on sociodemographic, clinical-laboratory and therapeutic variables was made available on the SBN website on the period from August to December 2018. Participation in the census is voluntary and all dialysis centers were invited, by letter and e-mail, to answer the questionnaire and send their data electronically to SBN. After the initial invitation, new reminders were sent monthly to those who had not filled in their data by the collection deadline (December 31, 2018). During the survey period, SBN regional presidents were tasked with contacting the directors of dialysis centers in their respective regions and encouraging them to participate in the census. At the end of the data collection period, the SBN board of directors again contacted the dialysis centers, and emphasized the importance of participation.

Data on mortality rates and incident patients on dialysis were collected in July 2018 and estimated for the year. We grouped the data presented by the centers so as not to portray individual patient information. For the estimation of data at a national level, non-participating centers were assigned the average number of expected patients. For the rest of the variables, averages were also assigned based on the center’s geographical region.

To carry out the prevalence and incidence calculations, we obtained the data from the Brazilian Institute of Geography and Statistics (IBGE), based on the Brazilian population of July 2018, and the relevant data from different regions of the country. According to this institute, the Brazilian population in July 2018 was 208.49 million inhabitants. To estimate the proportion of patients who did not reach the recommended target^s^ 9 ^-^ 11 for the dialysis dose (Kt/V or urea reduction rate) we used serum levels of albumin, phosphorus, parathyroid hormone (PTH) and hemoglobin. Most of the data is descriptive and refers to 2018, some of which was compared with data from previous years.

### CALCULATIONS FROM ESTIMATES

Estimated total number (N) of patients on July 1: N of patients in the sample/proportion of participating centers. Estimated global prevalence: estimated total N of patients on July 1/Brazilian population on July 1 of the corresponding years, expressed in millions of inhabitants (pmp). In the regional and state estimates of N and ratios, the data considered was restricted to specific regions or states. Estimated total N of patients starting treatment in the corresponding years: (N informed of individuals starting treatment in July x 12)/proportion of active participating centers. Estimated global incidence: estimated total N of patients starting treatment/Brazilian population on July 1 in the corresponding years, expressed in pmp.

The prevalences related to demographic, clinical, laboratory and medication variables were expressed in relation to the totals derived from the responses related to each of the factors investigated among the 48,596 patients treated at the participating centers. Estimated total number of deaths in the corresponding years: (N of deaths reported in July x 12) / proportion of active participating centers. Crude mortality rate: Estimated total N of deaths in 2018 / Estimated N of dialysis patients on July 1 of the corresponding year.

## RESULTS

**Figura 1:**
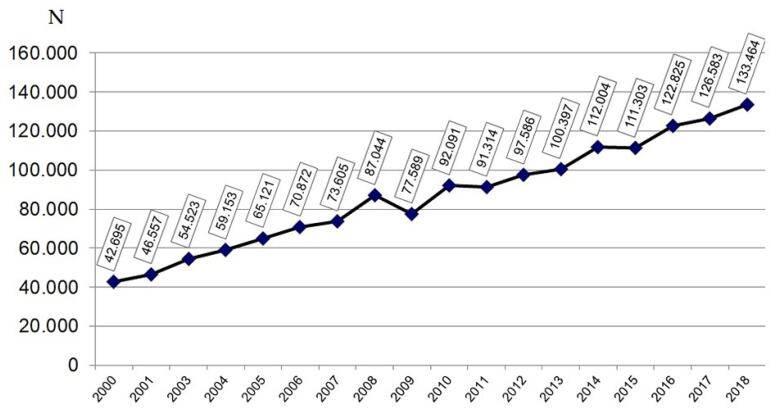
Número estimado de pacientes em diálise crônica por ano.

Analyzing the data from the 2009, 2013 and 2018 censuses comparatively, there was a progressive increase in the number of centers that maintained active chronic dialysis programs (594, 658 and 786, respectively), characterizing an increase of 32.3% during the decade. There has been a reduction in response to the census in recent years, both in percentage and absolute numbers. In 2009, 437 (69.8%) of the centers collaborated with their data, increasing to 334 (50.8%) in 2013 and 288 (36.6%) in 2018. This reduction in adherence was seen in all regions, but mainly in the South, with 69% of responses in 2013 and 34% in 2018. Thus, there was a discreet, but progressive reduction in the number of patients whose information contributed to the data in the annual report: 53,816, 50,961 and 49,215, in the years 2009, 2013 and 2018, respectively. When comparing the extremes, the reduction was of the order of 8.6%. During this period, there was a progressive increase in the number of patients prevalent in a chronic dialysis program (Figure 1), corresponding to an average annual increase of 5,587 patients.

Regarding the profile of dialysis clinics, evaluating data from 2009, 2013 and 2018, there is a predominance of private (70-72%), non-university (86-88%) clinics, with an increase in the percentage of satellite clinics (48-52%) and maintaining the public healthcare system (SUS) as the main paying source (80% in 2018). There was stability both in the distribution of clinics by region (half of them located in the Southeast) and in their occupancy rates (85-86%). Comparing data from 2013 and 2018, the centers that responded to the census reported having 1,863 nephrologists who worked in such clinics (a slight increase of 3.2%), maintaining the pattern of concentration in the Southeast, where there are 50% of these professionals. There was a slight reduction in the average number of patients per nephrologist (from 28 to 26); and the North region, despite the reduction (45 to 33), remains as the region with the largest number of patients/nephrologist. There was an increase in the number of clinics serving patients with acute renal failure (67% to 75%) and in conservative treatment of chronic kidney disease (73% to 84%). Regarding machine use time, there was an increase of 12% in the frequency of equipment with more than 6 years of use (32 to 44%), to the detriment of the reduction of those with 1-6 years of age (49% to 47%) and less than 1 year (16% to 9%).

**Figura 2:**
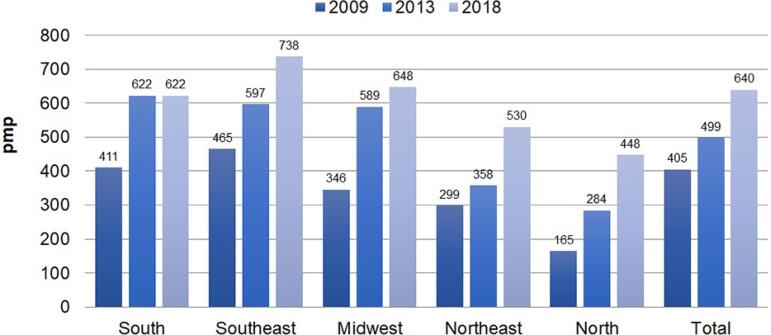
Evolução da prevalência estimada de pacientes em diálise por região geográfica no Brasil, 2009-2018 (por milhão da população).

**Figura 3:**
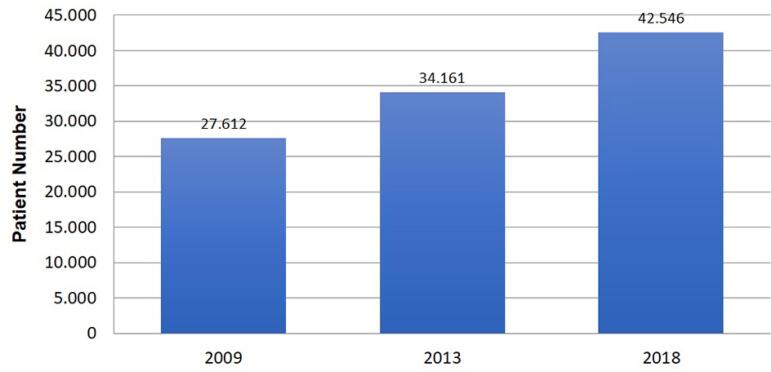
Incidência anual estimada de pacientes em diálise.

The estimated global prevalence of patients on chronic dialysis went from 405 pmp in 2009 to 640 pmp in 2018, corresponding to an absolute increase of 58%, with an average increase of 6.4% per year. Prevalence rates have increased progressively in all regions, except in the South, which has been stable since 2013 (Figure 2). The estimated number of new dialysis patients in 2018 was 42,546, an increase of 54.1% compared to 2009 (Figure 3). There was also an increase in the estimated incidence rate, which was 204 pmp in 2018, 20% higher than that reported for 2013. Table 1 provides data on the estimated incidences and prevalences by state of dialysis patients in the year 2018. The states with the highest estimated prevalence rates of dialysis patients were the Federal District, Rondônia and Alagoas, with 931, 874 and 865 pmp respectively; with the lowest rates recorded in Amazonas, Paraíba and Maranhão, with 313, 311 and 276 pmp, respectively. Hemodialysis remains the predominant renal clearance method, currently used for 92% of patients with end-stage renal disease (ESRD) (3% increase compared to 2009). As for peritoneal dialysis, there was a progressive reduction in the percentage of patients undergoing the method, with 10.5%, 9.2% and 7.8%, in 2009, 2013 and 2018, respectively. For the first time, there is a higher percentage of PD patients reimbursed by the SUS in relation to health insurance plans (7.8% vs. 7.0%, respectively). In this type of therapy, the predominance of Automated Peritoneal Dialysis remains, which corresponds to 5.7-5.8% of the total of patients, followed by Continuous Ambulatory Peritoneal Dialysis (CAPD) (1.9%). The reduction in the percentage of patients on peritoneal dialysis over time was mainly due to the reduction in the number of patients on CAPD, with a decrease from 3.5% to 1.9%.

**Table 1:** Estimated incidence and prevalence rates of dialysis patients per state in 2018

State	Incidence/pmp	Prevalence/pmp
AC	*	*
AL	383	865
AM	41	313
AP	*	*
BA	210	576
CE	120	579
DF	350	931
ES	*	638
GO	171	471
MA	78	276
MG	264	791
MS	183	755
MT	299	555
PA	80	418
PB	135	311
PE	158	523
PI	*	*
PR	211	680
RJ	217	856
RN	190	661
RO	191	874
RR	*	*
RS	218	618
SC	176	485
SE	*	*
SP	188	676
TO	*	*

**Note** (*estimates not done for lack of sufficient data).

**Figura 4:**
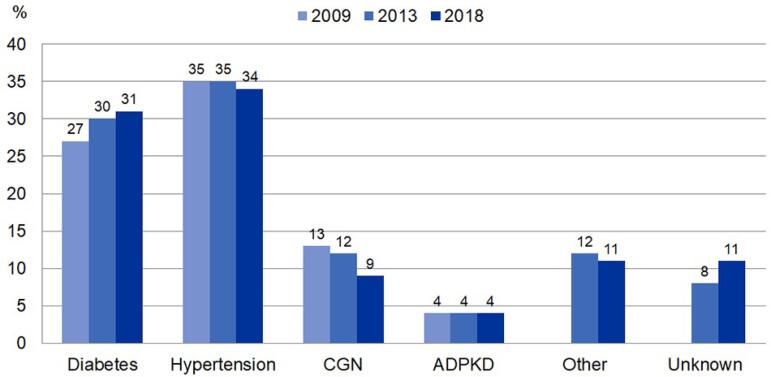
Distribuição de pacientes em diálise de acordo com doença de base, entre 2009-2018.

**Figura 5:**
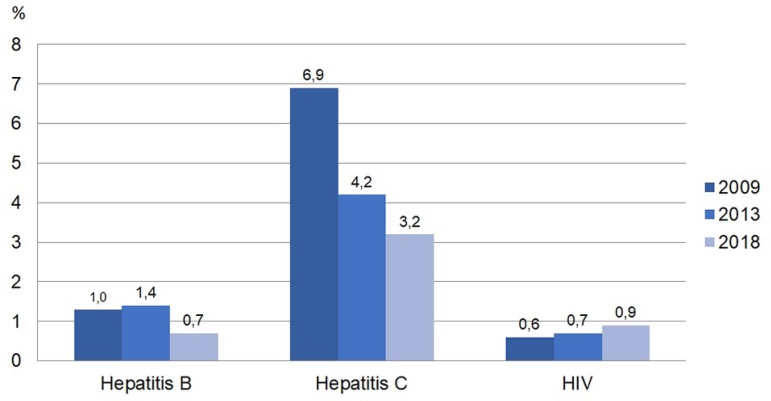
Prevalência de pacientes com sorologia positiva para vírus da Hepatite B, C e HIV.

**Figura 6:**
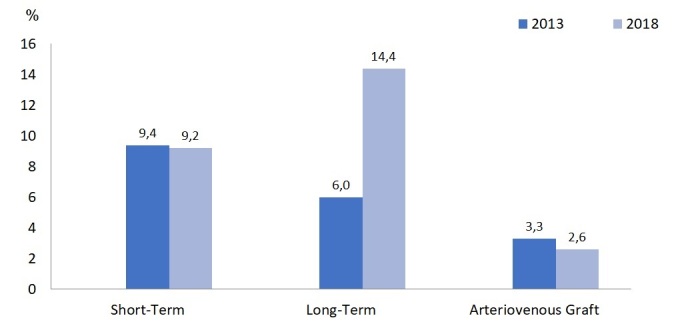
Distribuição dos acessos vasculares para hemodiálise entre 2013-2018.

**Figura 7:**
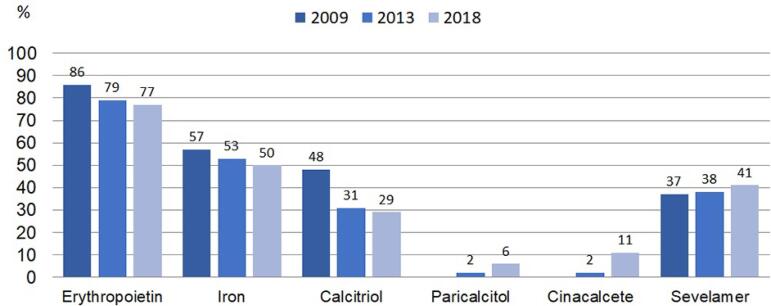
Percentual de pacientes em uso de medicações para tratamento de doença renal crônica em estádio terminal.

**Figura 8:**
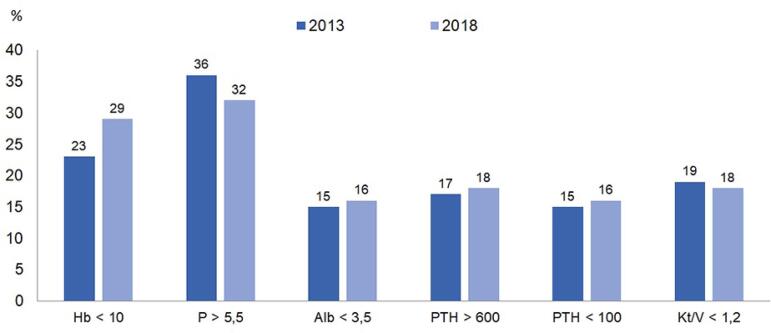
Proporção de pacientes com exames em não conformidade com índices recomendados pelo KDIGO.

**Figura 9:**
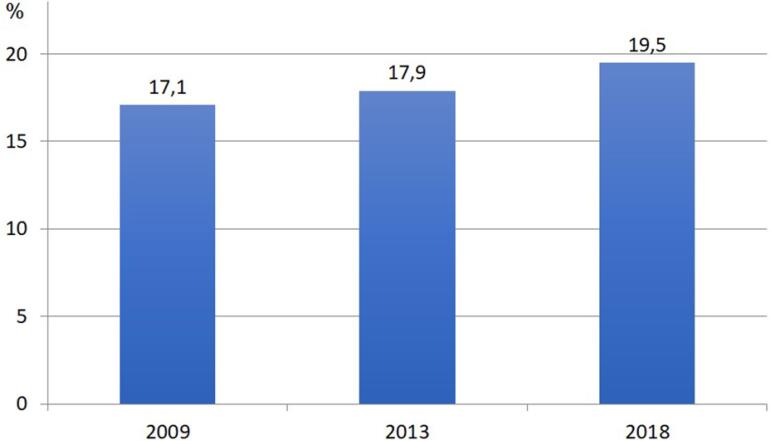
Taxa de mortalidade bruta anual estimada de pacientes em diálise.

Regarding the profile of dialysis patients, there is a predominance of males (58%); the majority in the age group between 45-64 years (41.5%), and over 65 years (35%). There was a percentage increase of about 4% in cases of diabetes-related kidney disease, a 4% reduction in cases of chronic glomerulopathy and an increase of 3% in those with undefined etiology (Figure 4). Regarding body mass index (BMI), about half (51%) of the patients have an adequate BMI (18.5-24.9 kg/m^2^), 8% below 18.5 kg/m^2^ and 41% with overweight/obesity (BMI ≥ 25 kg/m^2^). As for the positivity of dialysis patients with positive viral serologies, there was a reduction in the percentage of patients with serology for hepatitis B and particularly C viruses (with reductions greater than 50% compared to 2009 data) and stability in the proportion of patients with HIV (Figure 5). Concerning vascular access, the number of patients using a long-term catheter more than doubled when compared to 2013, with a reduction in the number of prostheses and stability in the number of patients with short-term catheters (Figure 6). Regarding the use of medications inherent to the treatment of end-stage CKD, there is a reduction in the use of erythropoietin, iron and calcitriol and an increase in the use of paricalcitol, cinacalcet and sevelamer (Figure 7). Regarding dialysis adequacy indicators based on the indices recommended by KDIGO, there was an increase in the proportion of patients with hemoglobin values below the goals recommended by KDIGO and in the proportion of patients with PTH values below 100 pg/mL, keeping the other indexes stable (Figure 8). The estimated number of patients on the waiting list for kidney transplantation decreased slightly, from 30,419, 31,351 to 29,545, in the 3 years considered, with a drop of 2.9% in the period. In 2018, the number corresponded to 22.1% of dialysis patients. In contrast, the estimated absolute number of deaths increased, from 13,235, 17,944 to 25,986 in 2008, 2013 and 2018, respectively. Figure 9 illustrates the estimated gross mortality rate comparing the three years, with an increase of 2.4%. Comparing only data available in the 2013 and 2018 censuses, the percentage of hospitalized patients per month was stable (5.8%). A question inserted from 2018 census shows that, in 78% of centers, the concentration of bicarbonate in the dialysis bath is the same for all patients, with a national median value of 32mEq/L.

## DISCUSSION

Since 1999, the Brazilian Society of Nephrology has been annually collecting data for the Brazilian Dialysis Census,^1^^-^ 8 whose objective is to present an overview of the profile of patients and dialysis clinics, focusing on aspects of the dialysis method, profile of clinics and patients, in addition to analysis of data related to adequacy in dialysis, vascular access and mortality, among others. Despite the huge importance of the census and the ease of completing it online only once a year, we have seen a reduction in the centers’ compliance in recent years, with only 36.6% of them contributing data in 2018.^1^^-^ 8

As in the Brazilian scenario, there is a worldwide trend of increasing the number of patients on dialysis, as well as prevalence rates.^12^^,^ 13 The incidence rate has shown greater variation, with a stable trend in some countries, increasing or decreasing in others.^13^^,^ 14 Estimates indicate that in 2010 there were about 2 million dialysis patients in the world and this figure should double by 2030.^12^ Data from 2018 from the Latin American Society of Nephrology and Hypertension (SLAHN)^14^ shows that the mean prevalence rate of patients on renal replacement therapy, RRT (including dialysis and transplantation), in Latin America was 805 per million people (pmp), with the highest rates seen in Puerto Rico, Chile and Mexico (2,129, 1,541 to 1,405 pmp, respectively). 2017 data from the United States Renal Data System (USRDS)^13^ show a prevalence rate of 2,203 pmp. In this context, Brazil has intermediate RRT rates, currently estimated at 876 pmp (including dialysis patients and those with functioning kidney transplantation), with the highest values ​​in the Southeast and Midwest regions. Regarding the annual incidence rate of dialysis patients in Brazil in 2018, the figure was 204 pmp, with higher values ​​in the South and Midwest regions. This rate is higher than the global rate in Latin America (154 pmp), but lower than in other Latin American countries, such as Puerto Rico, Mexico, Honduras and El Salvador (419, 344, 233 and 217, respectively), and the United States (370 pmp). In the latter country, there has been a trend towards the stability of incidence rates in recent years. Regarding dialysis, hemodialysis is the method most adopted in Brazil (92.2%), as well as in the United States (89.9%). The percentage of patients on peritoneal dialysis has been decreasing in both Latin America and the United States, but it still remains an important method, reaching figures close to 50% in countries like El Salvador and Guatemala, in contrast to Brazil and the USA, which have figures lower than 10%.^13^^,^ 14

Regarding the profile of prevalent patients on dialysis, males predominate, but there is a global trend of progressive increase in the age group of patients, with a significant percentage being.^12^^-^ 14 This fact can be explained by the increase in life expectancy among the general population, in addition to the progressive improvement of dialysis techniques and medications to support the complications of end-stage renal disease, also allowing greater longevity for prevalent patients. However, the increase in the percentage of long-lived patients implies an increase in the burden of comorbidities among patients undergoing renal replacement therapy. The increase in the number of elderly patients with renal functional failure associated with a greater presence of comorbidities has led to the discussion about the need for care planning and dialysis treatment in this population.^15^^,^ 16

About the underlying disease, unlike the USA and most countries in Latin America where diabetes-related renal disease is the main cause of ESRD,^13^^,^ 14 Brazil maintains hypertension as the main underlying cause, with stable figures for a few years, closely followed by those with kidney disease from diabetes. In our survey, we found an increase in the number of patients with venous catheters (from 6% to 14% for long-term catheters between 2013 and 2018). In the current Brazilian scenario, we know that this fact may be associated with greater difficulty in the availability of a vascular surgeons for making vascular access, since most clinics are predominantly funded by the SUS. Failure to make fistulas increases the incidence of patients with failure of vascular access and/or exceptional vascular accesses (such as transhepatic or translumbar catheters), in addition to prioritization for transplantation. With the progressive growth of the Interventional Nephrology field in Brazil, a specific training program for the preparation of arteriovenous fistula by the nephrologist during medical residency could contribute to the a in the number of patients using catheters for hemodialysis. Despite the incentive programs for making fistulas in the USA, in 2017, 80% of patients still started dialysis treatment through a central venous catheter, with a slight increase in the percentage of fistulas over the years. However, prevalent North American patients reached about 63% using a fistula; 17.6%, prosthesis; and 19.5%, catheters.^12^^,^ 13 Regarding the parameters of hemodialysis adequacy, we found that the percentage of patients who did not reach the dose parameters (Kt/V), nutrition and markers of bone kidney disease remains stable; however, there was an increase in the percentage of patients with hemoglobin <10g/dL, parallel to the reduction in the percentage of patients using erythropoietin. Still in relation to medication, in recent years, new drugs for the treatment of bone mineral disease related to CKD, such as cinacalcet and paricalcitol, started to be funded by the SUS. Both drugs had an increased use, since cinacalcet can assist in the treatment of patients with severe hyperparathyroidism and/or hyperparathyroidism in patients with hyperphosphatemia, and paricalcitol is more selective for the absorption of intestinal calcium, being associated with a lower incidence of hyperphosphatemia.^11^ Concomitantly, we found a reduction in the use of calcitriol over time.

An interesting fact to note is the reduction trend in the prevalence of positive serology for hepatitis C virus among patients on dialysis. In developed countries, the prevalence of such patients reached up to 9%, with higher numbers in other underdeveloped countries; however, this reduction tendency is global.^17^^-^ 19 Such progressive reduction in Brazil can be explained by the reduction in the number of blood transfusions by use of erythropoiesis-stimulating agents, by prohibiting the reuse of dialyzers and lines for patients with positive serology for hepatitis C since 2014,^20^ and also by the recent accessibility to treatments with high cure rates.

Regarding the percentage of dialysis patients enrolled in the transplant queue, Brazil and Uruguay share the first position in Latin America, with 22%; however, the highest transplant rates occur in Mexico, with 79 pmp (in Brazil, this figure is of 28 pmp).^14^ In the USA, 63.4% of dialysis patients were enrolled in 2017. In addition, there has been a downward trend in the number of patients in line in recent years in this country, due to changes in the process of allocating kidneys and the increase in the absolute number of kidney transplants in that country.^13^

Regarding the crude mortality rate in the evaluated period, we found a slight increase, from 17.1% to 19.5%, which, however, remains between 15-20%/year, in accordance with data reported by other countries.^12^^-^ 14 This trend can be explained by the increase in age and burden of comorbidities of patients prevalent in recent years.

For the first time, we have information on the use of bicarbonate in the dialysis bath in the census, and we found that, in 78% of the units, the concentration of bicarbonate in the bath was the same for all patients.

The fact that the information obtained through the census derives from the voluntary completion of the survey, the grouping of patient data by dialysis center and the lack of validation of the responses sent, so that inferences from this study must be made with caution.

## CONCLUSIONS

The Brazilian dialysis census remains an important tool for the quantification of data on dialysis and the planning of public health care policies. The 2018 survey compared to 2013 and 2009 showed an increase in the incidence and prevalence rates of dialysis patients. Significant inequality persists between states and regions in relation to these estimates, suggesting limitations in access to treatment, in addition to associations with the development indexes of each region/state. The proportion of patients with diabetes-related kidney disease has increased. Mortality rates have increased slightly and the use of venous catheters in hemodialysis has increased. On the other hand, the positivity of serology for hepatitis C continues to decline. Our data offer important subsidies to improve treatment and establish public policies for the care of patients with CKD undergoing dialysis in Brazil.
